# An Association between Bilirubin and Diabetic Retinopathy in Patients with Type 2 Diabetes Mellitus: An Effect Modification by Nrf2 Polymorphisms

**DOI:** 10.2174/0115733998327164240923070313

**Published:** 2024-11-29

**Authors:** Qiwei Fan, Shanshan Feng, Jianrong Chen, Yan Zhang, Jixiong Xu, Jiancheng Wang

**Affiliations:** 1 Department of Endocrine and Metabolism, The First Affiliated Hospital, Jiangxi Medical College, Nanchang University, Nanchang, Jiangxi, China;; 2 Jiangxi Clinical Research Center for Endocrine and Metabolic Disease, Nanchang, Jiangxi, China;; 3 Jiangxi Branch of National Clinical Research Center for Metabolic Disease, Nanchang, Jiangxi, China

**Keywords:** Serum bilirubin, diabetic retinopathy, Nrf2 polymorphisms, antioxidant, total bilirubin, diabetic mellitus

## Abstract

**Introduction:**

Diabetic retinopathy (DR) is a persistent microvascular complication associated with diabetes, and it constitutes a significant cause of visual impairment and blindness.

**Aims:**

This study aimed to assess the correlation between serum bilirubin levels and the prevalence of DR in patients diagnosed with type 2 diabetes mellitus (T2DM). Additionally, we sought to establish whether the polymorphisms of Nuclear Factor E2-Related Factor 2 (Nrf2) might modify this relationship.

**Methods:**

A cross-sectional study was undertaken in Jiangxi, China, from May, 2012 to December, 2014. Serum bilirubin levels were assessed in 558 subjects, and the correlation between bilirubin and DR was analyzed using generalized linear models with a logit link. The study utilized odds ratios (OR) and 95% confidence intervals (CI) to evaluate the relationship, both with and without the consideration of clinical risk factors.

**Results and Discussion:**

There was a significant inverse association between serum total bilirubin (TBiL) and the risk of DR (per 1-μmol/L increment; OR, 0.89; 95% CI: 0.84-0.94). Accordingly, when TBiL was categorized into tertiles, individuals in tertiles 2 and 3 exhibited significantly lower risks of DR compared to those in tertile 1. The OR for these tertiles was 0.54 (95% CI: 0.34-0.87) and 0.31 (95% CI: 0.19-0.52), respectively. Moreover, a stronger inverse relationship between TBiL and DR was observed in individuals carrying the CC and AC genotypes compared to those with the AA genotype. The OR for individuals with the CC/AC genotype was 0.87 (95% CI: 0.82, 0.92), while that for the AA genotype was 1.17 (95% CI: 0.95, 1.45). This difference was statistically significant (*p* for interaction = 0.001).

**Conclusion:**

There was a significant inverse association between bilirubin and DR in participants with CC or AC genotype. However, this inverse association was not seen in AA genotype participants.

## 1 INTRODUCTION

Diabetic Mellitus (DM), a global public health concern, continues to escalate in prevalence and exacerbate multiple complications, thereby placing a substantial strain on our limited healthcare resources [1, 2]. One persistent microvascular complication associated with DM is diabetic retinopathy (DR), which significantly contributes to visual impairment and blindness [3]. Consequently, there is a heightened interest in identifying novel, modifiable risk factors to enhance the prevention of DR and mitigate the substantial disease burden it imposes.

In recent years, oxidative stress has been identified as a crucial pathogenic factor contributing to the development of diabetic vascular complications, including DR [4]. Poor glycemic control, chronic hyperglycemia, and metabolic disorders exacerbate oxidative stress, leading to glycoxidation and peroxidation. These processes can impair endothelial function, thereby contributing to the progression of diabetic complications [5-7]. There is mounting evidence suggesting that the development of DR is associated with an imbalance between the production of Reactive Oxygen Species (ROS) and the antioxidant defense system [4, 5].

Bilirubin, once considered a byproduct of heme catabolism, is now recognized for its potential as an endogenous antioxidant and anti-inflammatory agent under physiological conditions [8]. It is inversely correlated with vascular complications and all-cause mortality [9-11]. Our previous research demonstrated that elevated levels of bilirubin can protect against the progression of chronic kidney disease and the first stroke [12, 13]. Nuclear factor-erythroid 2 related factor 2 (Nrf2), a transcription factor, plays a pivotal role in combating oxidative stress [14]. Numerous single nucleotide polymorphisms (SNPs) have been identified within the Nrf2 gene. Specifically, the Nrf2-617C/A polymorphism (rs6721961) has been linked to oxidative stress and the risk of newly diagnosed type 2 diabetes mellitus (T2DM) [15, 16]. However, whether serum bilirubin alone or in combination with Nrf2 polymorphisms may affect DR remains inconclusive.

This study aimed to explore the correlation between serum bilirubin levels (total and direct) and the occurrence of DR among patients diagnosed with T2DM. Furthermore, we investigated if this relationship persisted when other potential confounding variables were considered.

## 2 MATERIALS AND METHODS

### 2.1 Subjects and Study Design

The underlying rationale and the detailed study design have been previously elucidated [17]. Briefly, this study retrospectively analyzed all patients diagnosed with T2DM who visited the Department of Endocrinology, Diabetes, and Metabolic Diseases at the First Affiliated Hospital of Nanchang University between May, 2012 and December, 2014. The diagnosis of T2DM was based on self-reported doctor diagnosis, the use of anti-diabetic medications, or a fasting glucose concentration of ≥7.0 mmol/L, as per World Health Organization criteria [18]. DR was identified by an ophthalmologist following a comprehensive eye examination. The fundus photographs of both eyes were scrutinized and graded to confirm the presence and severity of DR. This condition primarily encompasses nonproliferative and proliferative types. Nonproliferative DR is characterized by symptoms, such as microaneurysms, hemorrhages, exudates, or microvascular abnormalities. Proliferative DR, on the other hand, is marked by the development of new vessels and fibrosis. Ethical clearance for this study was provided by the ethics committee of the First Affiliated Hospital of Nanchang University (CDYFY2014009), and written informed consent was obtained from all participants.

### 2.2 Inclusion Criteria

The study was comprised of patients aged 18 years or older who were diagnosed with T2DM in accordance with the American Diabetes Association (ADA) criteria. All participants provided their informed consent and volunteered to participate in the study.

### 2.3 Exclusion Criteria

We excluded from our study: (1) patients diagnosed with Type 1 Diabetes Mellitus; (2) individuals with hepatobiliary alanine aminotransferase (ALT) or aspartate aminotransferase (AST) levels exceeding twice the upper limit of normal, those with tumors, acute heart failure, or any other severe chronic conditions; (3) patients exhibiting additional fundus lesions; and (4) cases where the doctor was unable to visualize the fundus due to refractive medium opacity.

### 2.4 Sample Size Calculation and Sampling Technique

The sample size for our study was determined using a method inspired by Kendall's criterion [19]. Kendall's criterion is often applied to estimate sample sizes in cross-sectional studies. It suggests calculating a sample size five to ten times the number of variables and includes an allowance for a potential loss rate of 10%-20% during the study. Our study considered 17 variables, encompassing factors, such as age, gender, serum bilirubin levels, genetic polymorphisms, height, weight, smoking status, systolic blood pressure (SBP), diastolic blood pressure (DBP), fasting plasma glucose (FPG), glycated hemoglobin (HbA1c), total cholesterol (TC), low-density lipoprotein cholesterol (LDL-C), high-density lipoprotein cholesterol (HDL-C), triglyceride (TG), alanine aminotransferase (ALT), and aspartate aminotransferase (AST). To determine our sample size, we multiplied the number of variables by ten, resulting in 170 cases, and added an additional 20% to account for potential loss, totaling 34 cases. This calculation led us to a required sample size of 204 cases. To ensure a robust sample size, we ultimately included 518 cases in our investigation.

### 2.5 Collection of Demographic, Medical, and Laboratory Data

In this hospital-based cross-sectional study, we collected basic demographic data from medical records, including age, sex, height, weight, smoking status, SBP, and DBP. Body mass index (BMI) was calculated by dividing weight in kilograms by the square of height in meters. Blood and urine samples were collected on admission and used for analysis during the study period. We measured FPG, HbA1c, TC, LDL-C, HDL-C, TG, ALT, AST, total bilirubin (TBiL), and direct bilirubin (DBiL) levels using automatic clinical analyzers at the laboratory of the First Affiliated Hospital of Nanchang University, Nanchang, China.

### 2.6 Genotyping

For genotype analysis, genomic DNA was extracted from the peripheral blood leukocytes of each individual using a DNA isolation kit (Bioteke, Beijing, China). The genomic DNA was subjected to a polymerase chain reaction (PCR) with the following primers: Primer-F: 5ʹ-GGTTCCCGTTTTTCTCCC AGCTCT-3ʹ, Primer-R: 5ʹ-GGGTGGGGGGGGCTAAAG ATTT-3ʹ. PCR was performed in a 20 μL reaction volume containing 150 ng of template DNA, 0.2 μmol/L of forward and reverse primers, 0.225 U Taq DNA polymerase, 200 μmol/L of each deoxynucleotide phosphate, and 2.0 mmol/L Mg^2+^ on the ABI 7300 PCR system (Applied Biosystems, Foster City, CA, USA). The amplification protocol consisted of 94°C for 5 min, followed by 35 cycles of denaturation at 94°C for 20 s, annealing at 58°C for 30 s, and extension at 72°C for 30 s, with a final extension at 72°C for 5 min. The PCR products were randomly selected for DNA sequencing analysis by Nanjing GenScript Biotech Corporation.

### 2.7 Statistical Analysis

Continuous variables were analyzed using a t-test and are presented as the mean ± standard deviation (SD). For categorical variables, a chi-square test or Fisher’s exact test was used, and data are presented as percentages. The association between serum bilirubin and DR was evaluated using linear regression models (odds ratio (OR) and 95% confidence intervals (CI)) with and without adjustments for age, sex, BMI, smoking status, HbA1c, TC, and SBP. In addition, possible modifications of the association between serum bilirubin (per 1-μmol/L increment) and DR were assessed for the following variables: age (<60 *vs*. ≥60 years), sex, TC (<5.2 *vs.* ≥5.2 mmol/L), current smoking status (no *vs.* yes), and SBP (<140 *vs*. ≥140 mm Hg). Heterogeneity across subgroups was assessed using linear regression models and presented in forest plots, and interactions between subgroups and serum bilirubin concentrations were examined by likelihood ratio testing.

A two-tailed *p*-value <0.05 was considered statistically significant in all analyses. R software version 3.5.0. (http://www.R-project.org) was used for all statistical analyses.

## 3 RESULTS

### 3.1 Clinical Characteristics of the Participants

A total of 558 subjects were enrolled in the study. The study participants were categorized based on the tertiles of their baseline TBiL concentrations. Table **1** displays the general characteristics of the participants, organized by TBiL tertiles. There was a direct correlation between baseline TBiL concentrations and gender, baseline DBP, and levels of liver enzymes. Conversely, baseline TBiL concentrations demonstrated an inverse relationship with LDL-C.

The demographic and clinical characteristics of the DR and non-diabetic retinopathy (non-DR) groups are detailed in Table **S1**. The average age was 59.9 ± 10.7 years, with 51.6% of subjects being male. The average serum total bilirubin concentration was 8.8 (SD: 4.6) μmol/L. Subjects with DR exhibited significantly lower age and lower levels of ALT, TBiL, and DBiL compared to those without DR (Table **S1**).

### 3.2 Independent Effect of Serum Bilirubin and Nrf2 Polymorphisms on DR

A significant inverse relationship was observed between TBiL and DBiL levels and DR (Figs. **1A** and **1B**, respectively). For each 1-μmol/L increase in TBiL, there was an 11% decrease in the adjusted risk of DR, with an OR of 0.89 (95% CI: 0.84-0.94, *p* < 0.001). When TBiL was categorized into tertiles, the adjusted OR for DR was 0.54 (95% CI: 0.34-0.87, *p* = 0.016) for tertile 2 and 0.31 (95% CI: 0.19-0.52, *p* < 0.001) for tertile 3, relative to tertile 1 (Table **2**).

Similarly, a 1-μmol/L increase in DBiL levels was associated with a 20% reduction in the adjusted risk of DR, with an OR of 0.80 (95% CI: 0.70-0.91, *p* = 0.001). The adjusted OR for DR across DBiL tertiles was 0.69 (95% CI: 0.43-1.12, *p* = 0.134) for tertile 2 and 0.41 (95% CI: 0.24-0.68, *p* < 0.001) for tertile 3, compared to tertile 1 (Table **S2**).

However, genetic polymorphisms in Nrf2 did not significantly affect DR risk. The adjusted OR for DR among subjects with the AA genotype was 0.57 (95% CI: 0.27-1.20; *p* = 0.141) compared to those with the CC/CA genotype (Table **2**).

### 3.3 Stratified Analysis by Potential Effect Modifiers

Stratified analyses were conducted to more thoroughly evaluate the correlation between TBiL and DR across diverse subgroups. A significant interaction was identified between TBiL and Nrf2 polymorphisms in relation to DR (*p* for interaction = 0.001). Among subjects carrying the CC and AC genotypes, higher TBiL levels were significantly associated with a lower risk of DR (OR 0.87; 95% CI: 0.82, 0.92; *p* < 0.001). Among subjects carrying the AA genotype, however, higher TBiL levels had no significant effect on DR (OR 1.17; 95% CI: 0.95, 1.45; *p* = 0.147) (Table **3**).

None of the variables, including age (<60 *vs.* ≥60 years; *p* for interaction = 0.253), sex (*p* for interaction = 0.053), TC (<5.2 *vs.* ≥5.2 mmol/L; *p* for interaction = 0.483), current smoking status (yes *vs.* no; *p* for interaction = 0.988), and SBP (<140 *vs.* ≥140 mm Hg; *p* for interaction = 0.923) , significantly modified the association between TBiL and DR (Fig. **2**).

## 4 DISCUSSION

Our study uncovered an inverse correlation between serum bilirubin levels and the prevalence of DR, even after considering various confounding factors. This protective effect of bilirubin could be attributed to its antioxidant properties, which mitigate oxidative stress in the retinal vasculature. Furthermore, our research extends the results of prior studies by indicating that the association between bilirubin and DR can be significantly influenced by Nrf2 polymorphisms. Participants with elevated serum bilirubin levels and the CC or AC genotype exhibited a significant inverse correlation with DR.

The potential role of oxidative stress in the development of DR and the antioxidant and cytoprotective attributes of bilirubin have led to an escalating interest in the correlation between serum bilirubin concentration and the risk of DR. Some studies imply that elevated serum bilirubin might offer protection against DR [20, 21]. However, these claims require further substantiation, leaving the issue open to debate. Liu *et al.* [22] discovered a U-shaped association between baseline TBiL and DR incidence rather than a linear one. A study by Huang *et al.* [23] found no change in bilirubin levels in DM patients with varying durations of diabetes or the presence of DR. The discrepancy between our findings and those of previous studies could be partially attributed to variations in patient characteristics and research methodologies. It is important to note that potential effect modifiers were inadequately investigated in these studies. Our results suggest that the relationship between bilirubin and DR could be significantly influenced by NRF2 polymorphisms.

Hyperglycemia triggers the overproduction of superoxide radicals, which consequently disrupt the mitochondrial electron transport chain reactions. This process leads to the destruction of retinal endothelial and pericyte cells [24]. Furthermore, oxidative stress exerts a significant influence on various pathways and mechanisms. It can stimulate the production of advanced glycation end products and protein kinase C (PKC) while also activating the polyol pathway flux and the nicotinamide adenine dinucleotide phosphate-oxidase system. These actions exacerbate the adverse conditions [25-28]. Serum bilirubin, a potent endogenous antioxidant substance, exhibits a positive correlation with total antioxidant capacity [29]. It can effectively suppress overproduced reactive oxygen species (ROS), thereby affecting cell signaling and anti-inflammatory actions [30]. Our findings, coupled with previous research, reinforce the hypothesis that bilirubin may confer protective benefits in DR. This potential protective effect may be attributed to its antioxidant properties.

Nrf2, a key transcription factor, plays a role in upregulating antioxidant gene expression in response to oxidative stress [14]. Our study discovered a significant inverse correlation between bilirubin and DR levels in participants with the CC or AC genotype. This correlation was absent in those with the AA genotype. Suzuki *et al.* noted a decrease of approximately 40% in Nrf2 mRNA levels in A/A homozygotes for the rs6721961 SNP compared to C/A heterozygotes or C/C homozygotes. This implies that a homozygous substitution of A for C at rs6721961 significantly diminishes Nrf2 expression [31]. Wang *et al.* [16] reported that individuals with the AA genotype had significantly reduced antioxidant capacity compared to those with the CC genotype. Therefore, it is plausible that the ability of the Nrf2 transcription factor to modulate antioxidants plays a role in the Nrf2 pathway activation, potentially enhancing antioxidant capacity in participants with the CC or AC genotype.

Oxidative stress is a key factor in the development and progression of DR [4]. In diabetes, oxidative stress arises from an overproduction of ROS and a suppression of the antioxidant defense system, which is essential for maintaining cellular ROS balance [4, 5]. The interplay between bilirubin and the Nrf2 gene in DR risk is intricate. The antioxidant properties of bilirubin can modulate gene expression related to cellular defense against oxidative stress, particularly by activating the Nrf2/heme oxygenase-1 (HO-1) signaling pathway, which enhances antioxidant protein expression [32]. Conversely, activation of this pathway may increase bilirubin synthesis, potentially intensifying antioxidative effects [33, 34]. We hypothesize that bilirubin, in conjunction with the CC or AC genotypes that possess antioxidant capabilities, could synergistically boost the body's antioxidant defenses, possibly protecting against DR. However, the specific mechanisms connecting bilirubin and these genotypes to DR risk need further investigation.

Our study featured several unique aspects. Firstly, we found a significant modifying effect of Nrf2 polymorphisms on the correlation between serum bilirubin and DR. Consequently, it is essential to prioritize the examination of Nrf2 polymorphisms in conjunction with antioxidants in future studies. Secondly, prior research predominantly focused on the effects of TBiL on DR without differentiating between the various types of bilirubin. There have been limited studies that have explored both TBiL and DBiL concurrently. Our study suggests that both DBiL and TBiL have a protective effect against DR. However, this study also encountered potential limitations that should be taken into account when interpreting the results. Firstly, our study was cross-sectional, thereby limiting our capacity to establish a definitive causal link between serum bilirubin levels and the risk of developing DR. Secondly, our participants were exclusively drawn from university hospitals, implying that the findings may not be universally applicable to the general population or patients with T2DM in primary care settings. Consequently, further prospective studies with larger sample sizes are required to validate these observations.

## CONCLUSION

In conclusion, a significant inverse correlation was found between serum bilirubin concentrations and the prevalence of DR in participants with CC or AC genotypes. However, this correlation was not observed in participants with the AA genotype. Therefore, future prospective studies should confirm these findings.

## Figures and Tables

**Fig. (1):**
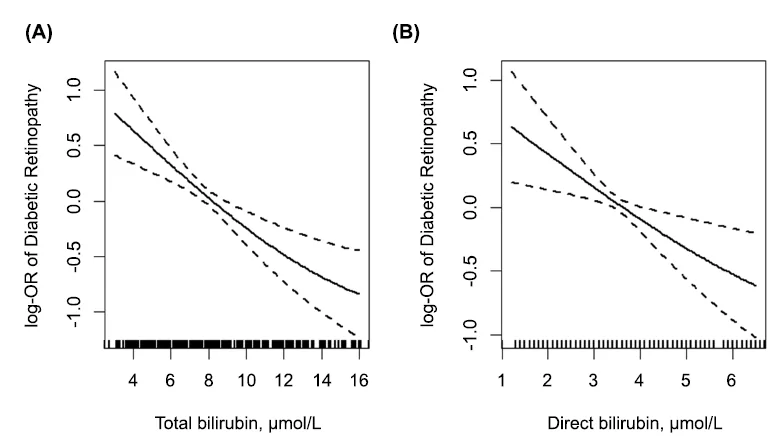
Association between TBiL (**A**) and DBiL (**B**) levels with DR in patients with type 2 diabetes mellitus. Adjusted for age, sex, body mass index, smoking status, HbA1c, total cholesterol, as well as systolic blood pressure.

**Fig. (2):**
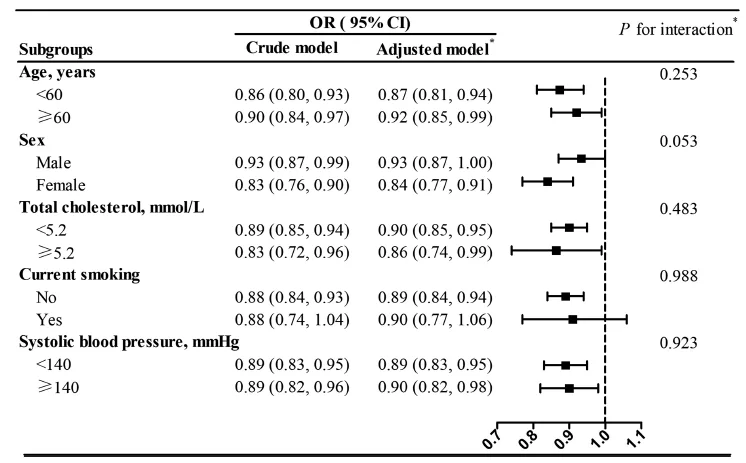
Association between serum TBiL and DR in various subgroups. Adjusted for age, sex, body mass index, smoking status, HbA1c, total cholesterol, as well as systolic blood pressure, if not stratified.

**Table 1:** Characteristics of the study participants according to TBiL concentrations or Nrf2 polymorphisms.

-	**TBiL, μmol/L**			***p* value**	**Nrf2 Polymorphisms**		***p*-value**
**Characteristics**	**Tertile 1 (<6.2)**	**Tertile 2 (6.2-9.5)**	**Tertile 3 (9.5-39.1)**		**CC/CA**	**AA**	
No. of participants	183	189	186	-	506	52	-
Age, year	60.0 (10.6)	61.2 (10.6)	58.6 (10.8)	0.067	59.8 (10.8)	61.6 (10.0)	0.236
Male, No. (%)	72 (39.3)	110 (58.2)	106 (57.0)	<0.001	266 (52.6)	22 (42.3)	0.159
BMI, kg/m^2^	23.2 (4.0)	24.5 (3.9)	24.7 (3.9)	0.015	24.2 (4.0)	23.6 (3.3)	0.494
Current smoker, No. (%)	22 (12.9)	27 (15.1)	15 (8.5)	0.151	61 (12.8)	3 (6.1)	0.174
**Blood Pressure, mm Hg**							
Systolic BP at baseline	135.1 (19.7)	135.7 (17.2)	134.3 (19.3)	0.779	135.1 (19.0)	135.0 (15.9)	0.97
Diastolic BP at baseline	80.2 (11.2)	84.2 (11.3)	84.1 (11.9)	<0.001	82.7 (11.7)	84.1 (10.7)	0.431
**Laboratory Results**							
Fasting glucose, mmol/L	8.3 (4.7)	8.4 (4.3)	8.4 (4.0)	0.976	8.5 (4.4)	7.7 (3.9)	0.256
HbA1c (%)	8.5 (2.5)	9.2 (2.5)	8.4 (2.2)	0.003	8.7 (2.4)	9.1 (2.9)	0.277
TC, mmol/L	4.3 (1.4)	4.5 (1.0)	4.2 (1.4)	0.06	4.3 (1.3)	4.1 (1.2)	0.205
Triglycerides, mmol/L	1.5 (1.1)	1.7 (1.8)	1.5 (1.4)	0.235	1.5 (1.3)	1.9 (2.6)	0.044
LDL-C, mmol/L	2.8 (0.9)	2.8 (1.4)	2.4 (0.9)	0.002	2.6 (1.1)	2.4 (1.0)	0.164
HDL-C, mmol/L	1.2 (0.5)	1.6 (7.8)	1.1 (0.3)	0.48	1.3 (4.7)	1.2 (0.7)	0.825
Alanine trasaminase	17.3 (8.8)	22.5 (11.5)	23.9 (12.2)	<0.001	21.2 (11.2)	22.2 (12.3)	0.552
Aspartate transaminase	19.7 (6.8)	21.3 (6.6)	22.0 (7.6)	0.006	21.0 (7.0)	21.6 (8.0)	0.55
Total bilirubin, μmol/L	4.7 (1.1)	7.8 (0.9)	13.8 (4.2)	<0.001	8.8 (4.6)	8.8 (4.4)	0.971
Direct bilirubin, μmol/L	2.5 (1.3)	3.4 (0.5)	5.6 (2.0)	<0.001	3.8 (1.9)	4.1 (2.4)	0.242

Note: *Continuous variables are presented as mean (standard deviation), and categorical variables are presented as numbers (percentages).**Abbreviations:** TBiL, total bilirubin; DR, diabetic retinopathy; BMI, body mass index; BP, blood pressure; TC, total cholesterol; LDL-C, low-density lipoprotein cholesterol; HDL-C, high-density lipoprotein cholesterol; HbA1c, hemoglobin A1c.

**Table 2:** The individual effect of TBiL concentrations (T1-T2 *vs.* T3) and Nrf2 polymorphisms (CC/CA, AA) on diabetic retinopathy.

**Outcomes**	**Events/N (%)**	**OR (95% CI)**		***p*-value**
		**Unadjusted**	**Adjusted**	
**TBIL**	-	-	-	-
Continuous (μmol/L)	558	0.88 (0.84, 0.93)	0.89 (0.84, 0.94)	<0.001
**Tertiles**				
T1 (<6.2)	82/183 (44.8)	Ref (1.00)	Ref (1.00)	-
T2 (6.2-9.5)	53/189 (28.0)	0.48 (0.31, 0.74)	0.54 (0.34, 0.87)	0.016
T3 (9.5-39.1)	36/186 (19.4)	0.30 (0.19, 0.47)	0.31 (0.19, 0.52)	<0.001
*p* for trend	-	<0.001	<0.001	-
**Categories**	-	-	-	-
T1-T2 (<9.5)	135/372 (36.3)	Ref (1.00)	Ref (1.00)	-
T3 (≥9.5)	36/186 (19.4)	0.42 (0.28, 0.64)	0.42 (0.27, 0.66)	<0.001
**NRF2 Polymorphisms**				
CC/CA	161/506 (31.8)	Ref (1.00)	Ref (1.00)	-
AA	10/52 (19.2)	0.51 (0.25, 1.04)	0.57 (0.27, 1.20)	0.141

**Note: ** *Adjusted for age, sex, body mass index, smoking status, HbA1c, total cholesterol, as well as systolic blood pressure. **Abbreviations:** TBiL, total bilirubin; OR, odds ratio; CI, confidence interval.

**Table 3:** The joint effect of baseline TBiL concentrations and Nrf2 polymorphisms (CC/CA, AA) on diabetic retinopathy.

-	**OR (95% CI)**		***p* value**	*p* for Interaction*
**Nrf2 Polymorphisms**	**Unadjusted**	Adjusted*		
CC/CA	0.86 (0.81, 0.91)	0.87 (0.82, 0.92)	<0.001	0.001
AA	1.12 (0.96, 1.30)	1.17 (0.95, 1.45)	0.147	-

**Note:** *Adjusted for age, sex, body mass index, smoking status, HbA1c, total cholesterol, as well as systolic blood pressure. **Abbreviations:** TBiL, total bilirubin; OR, odds ratio; CI, confidence interval.

## Data Availability

All data generated or analysed during this study are included in this published article.

## LIST OF ABBREVIATIONS

DM: Diabetic Mellitus
DR: Diabetic Retinopathy
Nrf2: Nuclear Factor E2 (NF-E2)-related Factor 2
SNPs: Single Nucleotide Polymorphisms
ALT: Alanine Aminotransferase
AST: Aspartate Aminotransferase
SBP: Systolic Blood Pressure
DBP: Diastolic Blood Pressure
BMI: Body Mass Index
FPG: Fasting Plasma Glucose
HbA1c: Glycated Hemoglobin
TC: Total Cholesterol
LDL-C: Low-density Lipoprotein Cholesterol
HDL-C: High-density Lipoprotein Cholesterol
TG: Triglyceride
TBiL: Total Bilirubin
DbiL: Direct Bilirubin
SD: Standard Deviation
OR: Odds Ratio
CI: Confidence Interval

## STANDARDS OF REPORTING

STROBE guidelines were followed.
