# Supplementary material for: An Association between Bilirubin and Diabetic Retinopathy in Patients with Type 2 Diabetes Mellitus: An Effect Modification by Nrf2 Polymorphisms
Source: Curr Diabetes Rev. 2024 Nov 29;22(4):E15733998327164. doi: 10.2174/0115733998327164240923070313 (PMC13711342; doi:10.2174/0115733998327164240923070313)
Supplement: Supplementary file 1 [file CDR-22-4-E15733998327164_SD1.pdf]

Supplementary Material

An Association between Bilirubin and Diabetic Retinopathy in Patients with Type 2 Diabetes Mellitus: An Effect Modification by Nrf2 Polymorphisms

Qiwei Fan[1,2,3,#], Shanshan Feng[1,2,3,#], Jianrong Chen[1,2,3], Yan Zhang[1,2,3], Jixiong Xu[1,2,3,*] and Jiancheng Wang[1,2,3,*]

[1]Department of Endocrine and Metabolism, The First Affiliated Hospital, Jiangxi Medical College, Nanchang University, Nanchang, China; [2]Jiangxi Clinical Research Center for Endocrine and Metabolic Disease, Nanchang, Jiangxi, China; [3]Jiangxi Branch of National Clinical Research Center for Metabolic Disease, Nanchang, Jiangxi, China

Table S1. Characteristics of the study participants without and with diabetic retinopathy[*].

Characteristics	Total	Without DR	With DR	*p* value
No. of participants	558	387	171	
Age, year	59.9 (10.7)	60.9 (11.1)	57.8 (9.5)	0.002
Male, No. (%)	288 (51.6)	179 (46.3)	91 (53.2)	0.129
BMI, kg/m^2	24.2 (3.9)	24.3 (4.1)	23.8 (3.5)	0.290
Current smoker, No. (%)	64 (12.2)	46 (12.6)	18 (11.1)	0.621
NRF2 polymorphisms				
CC	247 (44.3)	160 (41.3)	87 (50.9)	0.046
AC	259 (46.4)	185 (47.8)	74 (43.3)	
AA	52 (9.3)	42 (10.9)	10 (5.8)	
Blood pressure, mmHg				
Systolic BP at baseline	135.1 (18.7)	134.8 (18.7)	135.6 (18.8)	0.630
Diastolic BP at baseline	82.9 (11.6)	82.7 (11.9)	83.2 (11.0)	0.627
Laboratory results				
Fasting glucose, mmol/L	8.4 (4.3)	8.3 (4.3)	8.7 (4.4)	0.323
HbA1c (%)	8.7 (2.4)	8.7 (2.4)	8.8 (2.5)	0.667
TC, mmol/L	4.3 (1.3)	4.3 (1.2)	4.4 (1.4)	0.461

Triglycerides, mmol/L	1.5 (1.5)	1.6 (1.5)	1.5 (1.5)	0.593
LDL-C, mmol/L	2.6 (1.1)	2.6 (1.1)	2.6 (1.0)	0.689
HDL-C, mmol/L	1.3 (4.5)	1.4 (5.4)	1.2 (0.4)	0.627
Alanine trasaminase	21.3 (11.3)	22.2 (11.9)	19.3 (9.6)	0.005
Aspartate transaminase	21.0 (7.1)	21.3 (7.2)	20.5 (6.7)	0.201
Total bilirubin, μmol/L	8.8 (4.6)	9.4 (4.7)	7.4 (3.9)	<0.001
Direct bilirubin, μmol/L	3.8 (1.9)	4.0 (2.0)	3.3 (1.7)	<0.001

Note: *Continuous variables are presented as mean (standard deviation), categorical variables are presented as numbers (percentages). **Abbreviations:** DR, diabetic retinopathy; BMI, body mass index; BP, blood pressure; TC, total cholesterol; LDL-C, low density lipoprotein cholesterol; HDL-C, high density lipoprotein cholesterol; HbA1c, hemoglobin A1c.

Table S2. Association of serum DBiL levels and diabetic retinopathy.

		OR (95% CI)		
**Outcomes**	**No.**	**Unadjusted**	**Adjusted***	***P* value**
DBiL				
Continuous (μmol/L)	558	0.78 (0.68, 0.89)	0.80 (0.70, 0.91)	0.001
Tertiles				
T1 (<5.6)	137	1.00	1.00	
T2 (5.6-7.9)	136	0.62 (0.40, 0.96)	0.69 (0.43, 1.12)	0.134
T3 (7.9-10.9)	143	0.37 (0.24, 0.59)	0.41 (0.24, 0.68)	<0.001
P for trend		<0.001	<0.001	

Note: *Adjusted for age, sex, body mass index, smoking status, HbA1c, total cholesterol, as well as systolic blood pressure. **Abbreviations:** DBiL, direct bilirubin; OR, odds ratio; CI, confidence interval.
